# Reduced muscle strength in ether lipid‐deficient mice is accompanied by altered development and function of the neuromuscular junction

**DOI:** 10.1111/jnc.14082

**Published:** 2017-09-25

**Authors:** Fabian Dorninger, Ruth Herbst, Bojana Kravic, Bahar Z. Camurdanoglu, Igor Macinkovic, Gerhard Zeitler, Sonja Forss‐Petter, Siegfried Strack, Muzamil Majid Khan, Hans R. Waterham, Rüdiger Rudolf, Said Hashemolhosseini, Johannes Berger

**Affiliations:** ^1^ Department of Pathobiology of the Nervous System Center for Brain Research Medical University of Vienna Vienna Austria; ^2^ Section for Synapse Formation Center for Brain Research Medical University of Vienna Vienna Austria; ^3^ Center for Pathophysiology Infectiology and Immunology Medical University of Vienna Vienna Austria; ^4^ Institute of Biochemistry Friedrich‐Alexander University of Erlangen‐Nuremberg Erlangen Germany; ^5^ Institute of Toxicology and Genetics Karlsruhe Institute of Technology Eggenstein‐Leopoldshafen Germany; ^6^ Institute of Molecular and Cell Biology Faculty of Biotechnology University of Applied Sciences Mannheim Mannheim Germany; ^7^ Laboratory Genetic Metabolic Diseases Academic Medical Center University of Amsterdam Amsterdam The Netherlands; ^8^ Present address: Institute of Molecular Biology and Tumor Research (IMT), School of Medicine, Philipps University Marburg Hans‐Meerwein‐Str. 2, Biomedical Research Centre (BMFZ), 35043 Marburg Germany

**Keywords:** acetylcholine receptor, ether lipid, neuromuscular junction, peroxisome, plasmalogen

## Abstract

Inherited deficiency in ether lipids, a subgroup of phospholipids whose biosynthesis needs peroxisomes, causes the fatal human disorder rhizomelic chondrodysplasia punctata. The exact roles of ether lipids in the mammalian organism and, therefore, the molecular mechanisms underlying the disease are still largely enigmatic. Here, we used *glyceronephosphate O‐acyltransferase* knockout (*Gnpat* KO) mice to study the consequences of complete inactivation of ether lipid biosynthesis and documented substantial deficits in motor performance and muscle strength of these mice. We hypothesized that, probably in addition to previously described cerebellar abnormalities and myelination defects in the peripheral nervous system, an impairment of neuromuscular transmission contributes to the compromised motor abilities. Structurally, a morphologic examination of the neuromuscular junction (NMJ) in diaphragm muscle at different developmental stages revealed aberrant axonal branching and a strongly increased area of nerve innervation in *Gnpat* KO mice. Post‐synaptically, acetylcholine receptor (AChR) clusters colocalized with nerve terminals within a widened endplate zone. In addition, we detected atypical AChR clustering, as indicated by decreased size and number of clusters following stimulation with agrin, *in vitro*. The turnover of AChRs was unaffected in ether lipid‐deficient mice. Electrophysiological evaluation of the adult diaphragm indicated that although evoked potentials were unaltered in *Gnpat* KO mice, ether lipid deficiency leads to fewer spontaneous synaptic vesicle fusion events but, conversely, an increased post‐synaptic response to spontaneous vesicle exocytosis. We conclude from our findings that ether lipids are essential for proper development and function of the NMJ and may, therefore, contribute to motor performance.

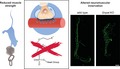

**Read the Editorial Highlight for this article on page** 463.

**Abbreviations used**
(m)EPP: (miniature) end plate potential
AChR: acetylcholine receptor
BTX: bungarotoxin
GNPAT: glyceronephosphate O‐acyltransferase
GPI: glycosyl‐phosphatidyl‐inositol
NMJ: neuromuscular junction
RCDP: rhizomelic chondrodysplasia punctata

In addition to a variety of proteins, also the lipid environment, particularly the different types of glycerophospholipids, has emerged as an important factor modulating synaptic activity (Rohrbough and Broadie 2005; Davletov and Montecucco 2010). Lipid bilayers and their interplay with the associated proteins provide the framework for endo‐ and exocytotic processes during the synaptic vesicle cycle regulating the release of neurotransmitters (Davletov and Montecucco 2010). The detailed molecular composition of the membranes involved has been shown to be of crucial importance for the regulation of the dynamics of fusion events in the nervous system (Chernomordik *et al*. 2006).

Ether (phospho)lipids constitute a particular subgroup of glycerophospholipids, which differs from the more abundant diacyl glycerophospholipids by the nature of the chemical bond attaching fatty acids to the *sn‐1* carbon of the glycerol backbone (diacyl phospholipids: ester bond; ether lipids: O‐alkyl, i.e., ether bond). Several subtypes of ether lipids have been identified: plasmalogens, the platelet‐activating factor, a potent inflammatory mediator (Prescott *et al*. 2000) or the lipid part of the glycosyl‐phosphatidyl‐inositol (GPI) anchor, a post‐translational modification that tethers proteins to the outer face of the cell membrane (Kanzawa *et al*. 2009). Plasmalogens, defined by a double bond adjacent to the O‐alkyl bond (vinyl ether bond), are the most abundant representatives, in humans making up about 20% of the total phospholipid mass. Inherited defects in ether lipid biosynthesis cause rhizomelic chondrodysplasia punctata (RCDP), which in its severest form is lethal in early childhood (Steinberg *et al*. 2006; Wanders and Waterham 2006). Affected patients are confronted with a variety of severe impairments, including growth and mental retardation, shortening of the proximal long bones, epiphyseal stippling, cataracts, facial dysmorphism, joint contractures, and respiratory problems (Braverman and Moser 2012). Although the genetic basis of RCDP is known, many of the underlying molecular processes remain unclear, as the functions of ether lipids have not been fully elucidated. Among the proposed tasks of plasmalogens are: the storage of essential polyunsaturated fatty acids like docosahexaenoic acid or arachidonic acid (Ford and Gross 1989); the stimulation of invariant natural killer T cells (Facciotti *et al*. 2012); and the generation of second messengers (Braverman and Moser 2012). Several *in vitro* studies have also proposed a role in antioxidative defense (Zoeller *et al*. 1999, 1988; Broniec *et al*. 2011), which has not yet been reliably confirmed *in vivo* (Brodde *et al*. 2012; Wallner and Schmitz 2011). Most importantly, as constituents of almost all biological membranes, plasmalogens shape membrane properties and structure as well as promote fusion and constriction processes (Thai *et al*. 2001; Hermetter *et al*. 1989; Paltauf 1994; Glaser and Gross 1994). Plasmalogens have been described to be enriched in lipid rafts (also termed membrane rafts) (Pike *et al*. 2002), small heterogeneous membrane domains, which are highly dynamic and compartmentalize cellular processes like signal transduction (Simons and Ikonen 1997; Pike 2006). This still expanding range of functions is reflected by the multifaceted pathogenesis of RCDP and other peroxisomal disorders, but alterations in the levels of ether lipids have also been reported in more common disease conditions (Berger *et al*. 2016), including Alzheimer's disease (Kou *et al*. 2011; Goodenowe *et al*. 2007), Parkinson's disease (Fabelo *et al*. 2011), Down syndrome (Murphy *et al*. 2000), or hypertension (Graessler *et al*. 2009).

The *glyceronephosphate O‐acyltransferase* knockout (*Gnpat* KO) mouse, in which the gene coding for the first enzyme in the ether lipid biosynthesis pathway (glyceronephosphate (or dihydroxyacetone phosphate) O‐acyltransferase, EC 2.3.1.42; Fig. 1) is disrupted, is a well‐established model of complete, isolated ether lipid deficiency (Rodemer *et al*. 2003) and, thus, serves as an ideal tool to study the biological relevance of this lipid family for various tissues in the context of a mammalian organism (Gorgas *et al*. 2006). *Gnpat* KO mice are characterized by a reduced, but highly variable lifespan, growth deficits, male infertility, and ocular anomalies (Rodemer *et al*. 2003; Saab *et al*. 2014; Komljenovic *et al*. 2009). More detailed studies of the central nervous system revealed abnormalities in cerebellar structures and in evoked neurotransmitter release from pre‐synaptic nerve terminals (Brodde *et al*. 2012; Teigler *et al*. 2009). Recently, deficits in myelination and Schwann cell development were demonstrated in the peripheral nervous system of *Gnpat* KO mice (da Silva *et al*. 2014).

**Figure 1:**
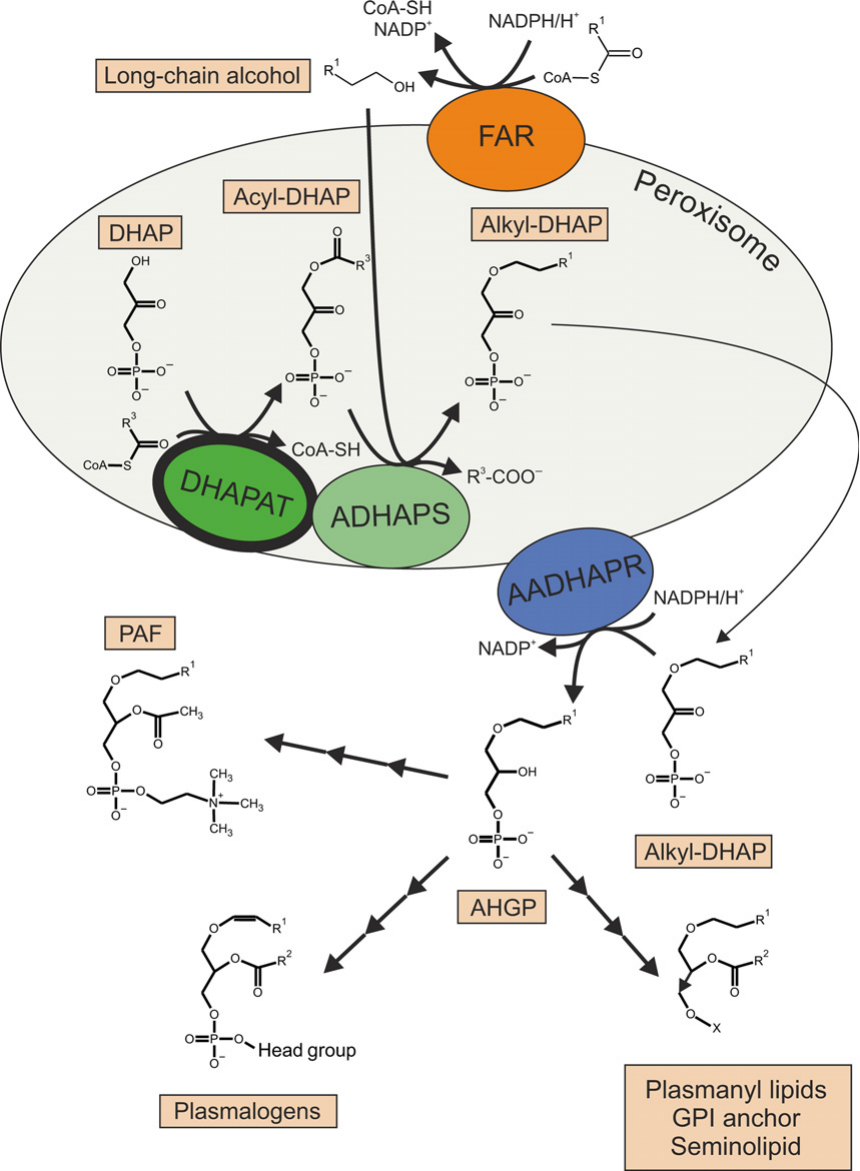
Overview of the peroxisomal contribution to ether lipid biosynthesis. Dihydroxyacetone phosphate O‐acyltransferase (DHAPAT; bold circle), encoded by the *Gnpat* gene, catalyzes the first step in the pathway. After export of the precursor (alkyl‐DHAP) from the peroxisome, residual biosynthesis steps take place elsewhere, in case of plasmalogens at the endoplasmic reticulum. AADHAPR, acyl/alkyl‐dihydroxyacetone phosphate reductase; ADHAPS, alkyl‐dihydroxyacetone phosphate synthase; AHGP, 1‐alkyl‐2‐hydroxy‐glycerophosphate; DHAP, dihydroxyacetone phosphate; FAR, fatty acyl‐CoA reductase; GPI, glycosyl‐phosphatidyl‐inositol; PAF, platelet‐activating factor.

The neuromuscular junction (NMJ), referring to the synapse between nerve and muscle, because of its large size, accessibility and relatively simple structure, is a widely studied model of peripheral synapses (Sanes and Lichtman 2001). At the NMJ, the neurotransmitter acetylcholine binds reversibly to ionotropic acetylcholine receptors (AChRs) on the surface of muscle fibers. A characteristic feature of the adult NMJ is the organization of the post‐synaptic membrane into invaginations (junctional folds) containing extremely dense clusters of AChRs next to a variety of auxiliary proteins, which enable the transmission of the electric signal (Shi *et al*. 2012). The NMJ differs from central synapses in the elaborate series of maturation steps, which require several weeks. In this process, the heparan sulfate proteoglycan agrin, which is released by the motor axon, plays a central role (Bezakova and Ruegg 2003). Post‐synaptically, agrin binds to LRP4, which interacts with the skeletal muscle receptor tyrosine protein kinase MuSK, thereby inducing the activation of MuSK (Kim *et al*. 2008; Zhang *et al*. 2008). MuSK kinase activity induces the clustering of AChRs via a complex post‐synaptic machinery involving the cytoplasmic linker protein rapsyn (Apel *et al*. 1997). AChR clusters form early in development but undergo considerable remodeling at later developmental stages. Although they appear as oval plaque‐like structures on a flat surface in newborn mammals, they adopt a more complex shape concomitant with formation of the junctional folds during post‐natal development (Marques *et al*. 2000).

Novel determinants for the correct maturation and synaptic function of the NMJ are still being found. Based on their (i) significance for signaling cascades like the AKT (protein kinase B) pathway (da Silva *et al*. 2014), (ii) involvement in membrane fusion and constriction events (Glaser and Gross 1994), and (iii) abundance in synaptic vesicle and pre‐synaptic membranes (Takamori *et al*. 2006; Hofteig *et al*. 1985), ether lipids may well modulate the development and activity of the NMJ. Therefore, in this study, we analyzed the consequences of ether lipid deficiency for formation, maintenance, and function of the NMJ. Based on the observation of an abnormal motor behavior phenotype of the *Gnpat* KO mouse, potentially involving NMJ deficits, we characterized the morphology and electrophysiological properties of NMJs in these mice.

## Materials and methods

### Mice

**Figure 2:**
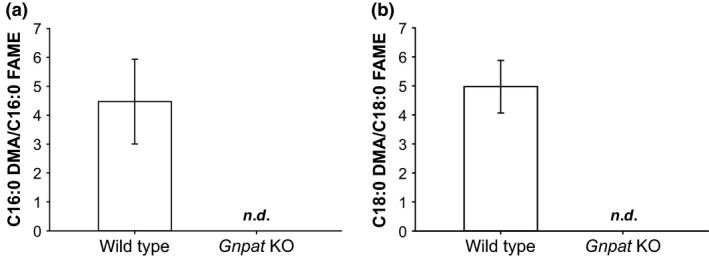
Absence of plasmalogens from muscle tissue of *Gnpat* KO mice. Plasmalogen levels were determined by detecting dimethylacetals (DMA) after acidic methanolysis and normalizing them to their corresponding fatty acid methyl esters (FAME). Neither C16:0 (a) nor C18:0 (b) DMA were detected in muscle tissue of *Gnpat* KO mice (WT, *n* = 4; *Gnpat* KO, *n* = 3). *n.d*., not detected

**Figure 3:**
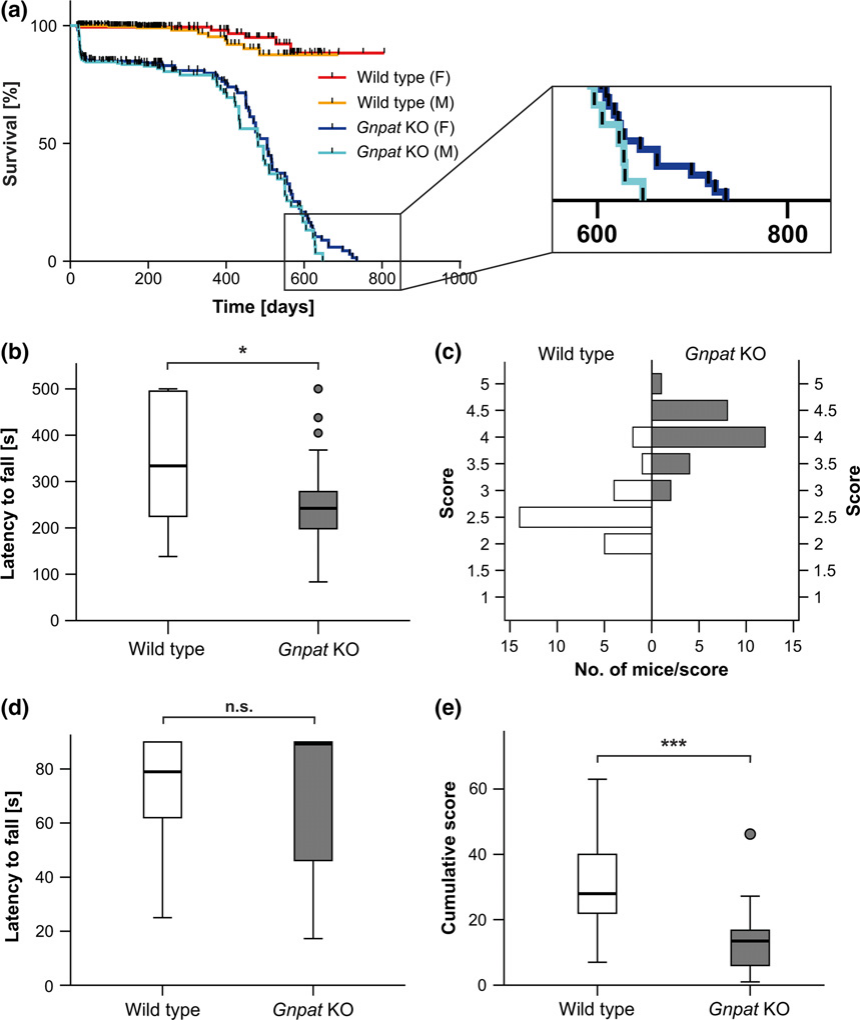
Motor impairment in ether lipid‐deficient mice. (a) Kaplan–Meier plots indicating survival functions of male (WT: *n* = 315, *Gnpat* KO: *n* = 244) and female (WT: *n* = 278, *Gnpat* KO: *n* = 203) mice. Note that some KO pups may have died before their birth was recorded. Statistical analysis using log‐rank tests revealed highly significant differences between the genotypes (*p *<* *0.001 for males, females or both sexes combined) but not between sexes, although the longest‐living animals were females (see inset). (b) In the accelerating rotarod test (maximal time 500 s), the latency to fall was recorded in adult WT (*n* = 26) and *Gnpat* KO (*n* = 27) mice. *p <* *0.05 (Mann–Whitney *U*‐test). (c) Scores obtained by the same cohort as in (b) in the balance beam test are depicted as the number of animals per genotype reaching a certain score (defined in Table S1); the better the performance, the lower the score. Statistical analysis using Mann–Whitney *U*‐test revealed a highly significant difference between the genotypes (*p *<* *0.001). (d) For the inverted screen task, the mean latency to fall was recorded for adult WT (*n* = 30) and *Gnpat* KO (*n* = 31) mice. *n.s*., not significant (Mann–Whitney *U*‐test) (e) Cumulative scores (time × weight coefficient) obtained in the weights test are depicted for the same cohorts as in (d). ***p <* *0.001 (Mann–Whitney *U*‐test). Box‐and‐whisker plots in (b), (d), and (e) are drawn according to Tukey's method with the horizontal, bold line indicating the median value.

*Gnpat* KO mice (*Gnpat* ^*tm1Just*^; MGI:2670462) were maintained on an outbred C57BL/6 × CD1 background (Rodemer *et al*. 2003); homozygous *Gnpat* ^*−/−*^ (KO) and *Gnpat* ^+/+^ (wild type, WT) littermates were obtained by mating heterozygous animals. To ensure complete ether lipid deficiency in the target tissue, we confirmed the absence of plasmalogens from dissected muscles (Fig. 2). In line with previous observations (Rodemer *et al*. 2003), *Gnpat* KO mice showed significantly decreased survival rates and the longest‐living animals were females (Fig. 3a). Slight deviations from the originally reported survival curves probably derive from a drift in the genetic background of the strain and attentive care of KO animals to ensure their survival. The *Gnpat* genotype was determined at weaning by PCR as described previously (Rodemer *et al*. 2003) and confirmed at the time of killing. Mice carrying a thermolabile variant of the simian virus 40 large tumor antigen under the mouse major histocompatibility complex H‐2K^b^ promoter (Tg(H2‐K1‐tsA58)11Kio, MGI:3762405, ‘Immorto’) (Jat *et al*. 1991) were crossed with *Gnpat* ^*tm1Just*^ mice for the establishment of immortalized myoblast cell lines. The presence of the transgene was determined at weaning or, in case of myoblast isolation experiments, after killing by PCR, as described previously (Kern and Flucher 2005). All mice were fed standard chow and water *ad libitum* and were housed in a temperature‐ and humidity controlled room with 12 : 12 h light–dark cycle and a low level of acoustic background noise at the in‐house animal facility (Medical University of Vienna). All animals received humane care and handling in compliance with institutional and national (Austrian) regulations (BGBl. II Nr. 522/2012) as well as the European Union Directive 2010/63/EU and the use of these genetically modified animals was approved (BMWF‐5.011/0003‐II/10b/2009). For all experiments with adult mice, age‐ and sex‐matched WT littermates were used as controls. For the studies of NMJ development, the sex of the fetuses was not determined. Staging of timed pregnancies was evaluated by vaginal plug detection (E0.5 = noon of the day following overnight mating, vaginal plug at morning inspection).

### Behavioral assays

Rotarod (Dumser *et al*. 2007), balance beam (Carter *et al*. 2001), weights, and inverted screen (Deacon 2013) tests were performed as described previously with slight modifications. Further information is provided in the Supporting Information and the adapted scoring system used for balance beam testing is specified in Table S1. Investigators were blinded to the genotype of the mice (although the phenotype of *Gnpat* KO mice is overt in most cases).

### Morphologic examination of NMJs

Staining of fetal diaphragms and adult muscles was performed as described previously (Herbst *et al*. 2002; Kravic *et al*. 2016). More detailed information is provided in the Supporting Information.

### Determination of AChR lifetime

Kinetics of AChR lifetimes in WT and *Gnpat* KO mice were analyzed by pulse labeling as described recently (Strack *et al*. 2011). Briefly, on day zero, mice were anesthetized by intraperitoneal administration of xylazin/zoletil and 10 μL ^125^I‐labeled bungarotoxin (BTX; Perkin Elmer) solution (2.5 μCi) were injected into *tibialis anterior* muscles of both hind limbs. During the following 24 days, measurements of emitted X‐rays from labeled muscles were performed under isoflurane narcosis (air mixture 0.6–1.5% administered by a tube) using a portable Germanium semiconductor counter (GX3018, Canberra Industries). Radiation emerging from the rest of the body was blocked by a lead shield with a circular fenestration positioned at the level of the *tibialis anterior* muscle. Individual measurements lasted for 300 s. Data were analyzed with the help of an attached multi‐channel analyzer (InSpector 2000 DSP Portable Spectroscopy Workstation, Canberra Industries). Obtained data were confirmed by microscopic determination of AChR stability as described (Roder *et al*. 2010).

### Monitoring neural transmission by electrophysiology

Recordings at the NMJ were mainly performed as described earlier (Kravic *et al*. 2016). Technical details can be retrieved from the Supporting Information. Quantal content was calculated from the ratio between end plate currents (EPCs) and miniature end plate currents (mEPCs). Capacitance was calculated as C = R/τ with C as capacitance, R as input resistance, and τ as the time constant of the voltage response to the current injection (84% of the rise time curve) based on previous literature (Gage and Eisenberg 1969). All electrophysiological recordings were performed at 23°C.

### Isolation and cultivation of primary myoblasts

Newborn mouse pups (*Gnpat* KO and WT carrying the H‐2Kb‐tsA58 transgene) were killed by decapitation and their limbs stored in growth medium (Dulbecco's modified Eagle's medium supplied with 50 units/mL penicillin, 100 μg/mL streptomycin, 10% fetal bovine serum (FBS), 10% horse serum, 0.5% (vol/vol) chick embryo extract). Muscle tissue was isolated and minced in phosphate‐buffered saline (PBS) with 1% glucose, taken up in growth medium and dissociated with 0.2% trypsin and 0.01% DNase for 30 min (37°C, 6% CO_2_). After centrifugation (5 min, 400 *g*), the pellet was dissolved in growth medium containing 20 U/mL recombinant mouse interferon‐γ (IFN‐γ, Peprotech) and a single‐cell solution prepared by use of pipet tips and cell strainers (100 μm, BD Falcon). Cells were pre‐plated for 35 min at 33°C, 6% CO_2_ and non‐adherent cells transferred to gelatin (0.2%)‐coated dishes. Individual clones of cells were expanded under the permissive conditions (33°C, 6% CO_2_) and their differentiation into myotubes induced by removal of IFN‐γ, chick embryo extract, and FBS as well as a shift of the growth temperature to 37°C. According to their ability to form myotubes, two *Gnpat* KO cell lines and one WT cell line were included in clustering experiments.

### AChR clustering assay

To induce AChR clustering, myotubes were stimulated with conditioned medium containing neural agrin (A4B8) prepared from HEK 293T cells (Herbst and Burden 2000; Tsim *et al*. 1992) for 8 h. To visualize surface AChRs, cells were fixed with 4% paraformaldehyde in PBS for 10 min at 23°C, washed twice with PBS for 5 min, and incubated with Alexa 594‐conjugated α‐BTX (200 ng/mL in 2% FBS/PBS) for 30 min. Cells were washed twice with PBS for 5 min and mounted in Mowiol 4‐88. AChR clusters were imaged with a DM‐IRB inverted fluorescence microscope (Leica, Wetzlar, Germany) using a 63X oil immersion magnification objective. Metamorph (Molecular Devices) and ImageJ software were used to acquire and quantify images as described previously (Camurdanoglu *et al*. 2016).

### Determination of plasmalogen levels

Plasmalogen levels in muscle homogenates were determined by detecting dimethylacetals as described previously (Dacremont and Vincent 1995). Additional information is provided in the Supporting Information.

### Statistical analysis

The groups of WT and *Gnpat* KO mice were compared with each other using two‐tailed Student's *t*‐tests or Mann–Whitney *U*‐tests, depending on the nature of the variable to be analyzed. Statistical details for each experiment can be found in the Figure legends. The number of animals was kept to an absolute minimum; where applicable, required numbers were estimated using Java applets assuming a minimal statistical power of 0.85.

## Results

### Ether lipid deficiency causes motor impairment

Observation of the home cage behavior of *Gnpat* KO mice revealed several features indicative of motor impairment such as unsteady gait. Therefore, we systematically assessed motor coordination and muscle strength in young adult mice in several behavioral tasks. In the accelerating rotarod test, *Gnpat* KO mice showed a significantly shorter latency to fall off the turning rod compared with WT mice (Fig. 3b). This difference is probably an underestimate, because some WT mice, after several training sessions, lost interest in the task and stopped running midway during the trial. Differences between WT and *Gnpat* KO mice were more obvious, when calculating the fraction of animals reaching the maximal time of the task (500 s) in at least one trial [WT: 11/26 animals (42%); *Gnpat* KO: 4/27 (15%)]. The same cohort of animals was subject to the balance beam task, during which the motor performance – in particular, foot placement and slips – while traversing a horizontal bar was evaluated. *Gnpat* KO animals experienced severe problems in this task, with their limbs regularly slipping below the horizontal midline of the bar. This resulted in significantly lower (i.e.*,* better) average scores (*p *<* *0.001, Mann–Whitney *U*‐test) ranging from 2 to 4 for WT and from 3 to 5 for *Gnpat* KO mice (Fig. 3c).

To more specifically address muscle strength, we applied two further simple tests: the inverted screen and the weights tests (Deacon 2013). In the inverted screen task, mice had to cling onto an inverted grid as long as possible or for a maximum of 90 s. In this rather unselective test, both WT and *Gnpat* KO mice performed similarly and many WT as well as KO animals reached the maximum time (Fig. 3d). The number of animals achieving the maximal time in at least one trial was 27/30 (90%) for WT and 23/31 (74%) for *Gnpat* KO mice. In contrast, the weights test, which assesses the weightlifting capability of mice, revealed significant differences between the genotypes (*p *<* *0.001, Mann–Whitney *U*‐test), with *Gnpat* KO mice achieving considerably lower scores than WT controls (Fig. 3e). Because ether lipid‐deficient animals are typically smaller than their WT littermates, we considered a normalization to the body weight for each mouse. However, correlation analysis within the genotype groups did not show a significant association between body weight and performance score (Fig. S1). To explore potential gender differences, we also exposed a considerable number of female mice (*n* = 18 per genotype) to the inverted screen and the weights tests in an independent series of experiments and obtained highly similar results as for males (data not shown). Taken together, our results indicate considerable motor behavior deficits, affecting motor coordination as well as muscle strength, in *Gnpat* KO mice.

### The lack of ether lipids impairs formation of the neuromuscular junction

**Figure 4:**
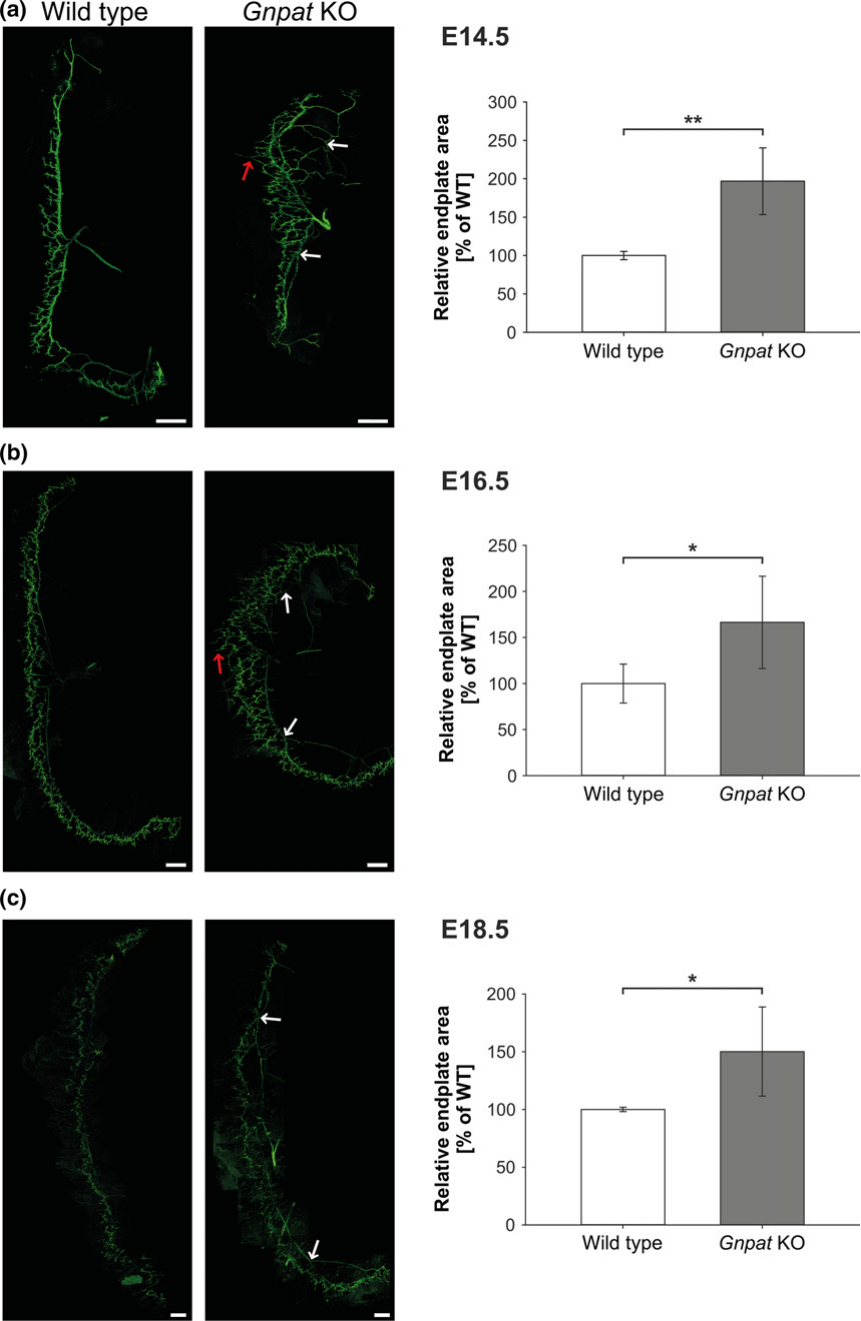
Wider area of nervous innervation and exaggerated phrenic nerve branching in ether lipid‐deficient diaphragm muscle. Whole mounts of diaphragms from WT and *Gnpat* KO fetuses were stained by indirect immunofluorescence with a mixture of antibodies specific for neurofilament M and synaptophysin and the area covered by neurons was quantified at E14.5 (WT, *n* = 4; *Gnpat* KO, *n* = 5) (a), E16.5 (WT, *n* = 4; *Gnpat* KO, *n* = 5) (b), and E18.5 (WT, *n* = 5; *Gnpat* KO, *n* = 8) (c). In *Gnpat* KO diaphragms, extensive branching of the main axons (white arrows) and excessive growth beyond the main axon (red arrows) are notable. For the mode of quantification, see Supporting Information. Bar charts to the right show the summary statistics (means ± SD) of genotype comparisons for each developmental time point. **p <* *0.01, *p <* *0.05 (two‐tailed Student's *t*‐test); scale bars = 250 μm (in each micrograph).

Although various parameters contribute to motor function, motor impairment has been widely associated with disturbances in the formation or the maintenance of the NMJ. Based on the observed behavioral phenotype, we, therefore, conducted a morphological examination of the NMJ in WT and *Gnpat* KO mice. Because of its thinness and accessibility, the diaphragm muscle is widely used to study motor innervation and NMJ morphology. Thus, we isolated diaphragms from WT and *Gnpat* KO mouse fetuses at different developmental stages (embryonic day (E) 14.5, 16.5, and 18.5) and visualized pre‐ and post‐synaptic components of the NMJ. At all time points examined, *Gnpat* KO diaphragms were characterized by abnormally extensive branching of the phrenic nerve and a general widening of the endplate zone (Fig. 4a–c). Main axons appeared partially defasciculated, particularly in the ventral region of the diaphragm, and we found a number of larger axons, each giving rise to several smaller branches (arrows in Fig. 4). Consequently, upon quantification, the area covered by nerves and nerve endings was significantly increased in *Gnpat* KO samples at all time points examined. Although this defect seemed less pronounced at a later embryonic stage (E18.5, Fig. 4c) – most likely because of the larger overall size of the diaphragm as well as the innervated area – we still observed considerably increased phrenic nerve branching also at this stage suggesting that the phenotype is not ameliorated during development. Concomitant with the alterations in nerve appearance, post‐synaptic AChR clusters on the muscle fibers were spread across a much wider area in *Gnpat* KO than in WT diaphragms (Fig. S2a). In spite of these abnormalities, there was a perfect colocalization of pre‐ and post‐synaptic components of the NMJ indicating that synapses are correctly assembled (Fig. S2b). Furthermore, we did not detect any apparent differences in the structure or the size of individual AChR clusters, which at the embryonic stage appear as oval plaques.

Upon maturation, rodent NMJs are pretzel‐like shaped (Sanes and Lichtman 2001). To judge whether mature AChR cluster formation is impaired in *Gnpat* KO mice in comparison with WT mice, we performed a quantitative analysis of AChR clusters in several hind limb muscles (*soleus*,* gastrocnemius*,* extensor digitorum longus,* and *tibialis anterior*). The morphometric analysis revealed a smaller volume and surface area of α‐BTX‐labeled AChR clusters in ether lipid‐deficient muscles (Fig. S3a–c). The effect of genotype on these two parameters was statistically highly significant for the *soleus* muscle. Also the difference in surface area in the *gastrocnemius* muscle reached statistical significance, whereas in the other muscles, only a trend was observed. There was no difference in the mean gray values of AChR clusters between genotypes indicating identical densities of AChRs (Fig. S3d). However, AChR clusters in *Gnpat* KO mice were clearly less fragmented than WT clusters in all four muscles analyzed (Fig. S3a and e).

**Figure 5:**
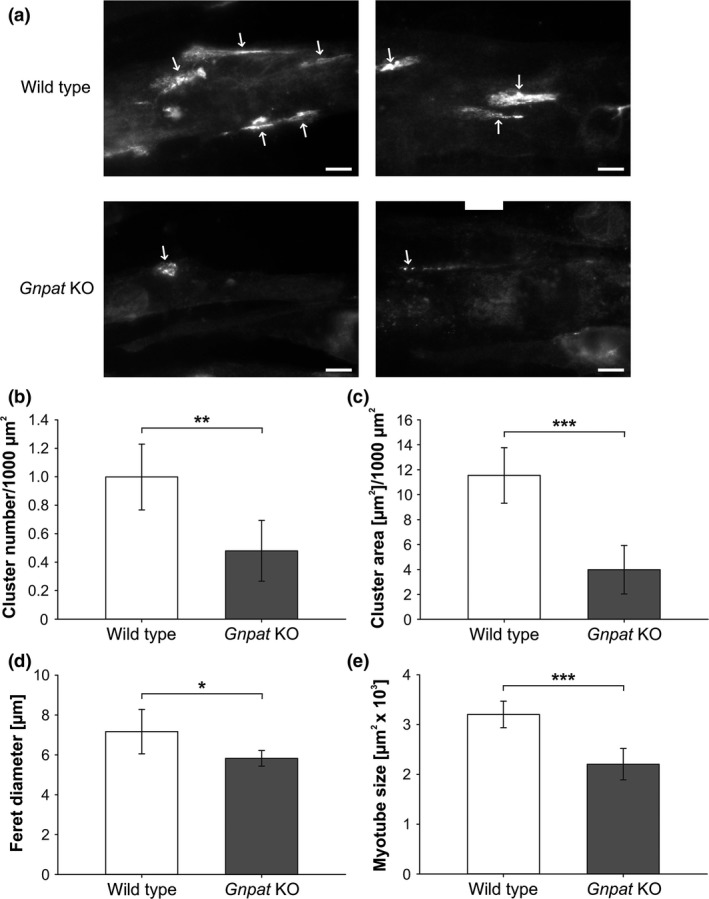
Impaired acetylcholine receptor (AChR) clustering *in vitro* in *Gnpat* KO myotubes. Clustering of AChRs was induced by stimulation with neural agrin in myotubes derived from immortalized myoblasts from WT and *Gnpat* KO mice. AChRs were stained with Alexa Fluor 594‐conjugated α‐BTX and representative images of clusters (indicated by arrows) are shown in (a) for both genotypes; scale bar: 10 μm. Quantifications of cluster number (b) and area (c), both normalized to myotube area, as well as the Feret diameter of clusters (d) and the size of myotubes (e) are presented as means ± SD (*n* = 5 experiments/genotype involving a total of > 225 clusters/genotype derived from one WT and two KO cell lines). ***p <* *0.001, **p <* *0.01, *p <* *0.05 (two‐tailed Student's *t*‐test).

To test whether an intrinsic clustering defect could account for these findings, we generated immortalized myoblasts and obtained two stable cell lines from *Gnpat* KO and one from WT mice carrying the *Immorto* (H‐2K^b^‐ts58) transgene. In order to assay AChR clustering, we stimulated differentiated myotubes derived from these myoblasts with neural agrin and analyzed the resulting patches of AChRs by fluorescence microscopy. AChR clustering was clearly impaired in ether lipid‐deficient myotubes, as indicated by significantly smaller and fewer AChR clusters per myotube area (Fig. 5a–d). Also the size of *Gnpat* KO myotubes themselves was decreased in comparison with WT tubes (Fig. 5e).

### AChR stability is not affected by ether lipid deficiency

**Figure 6:**
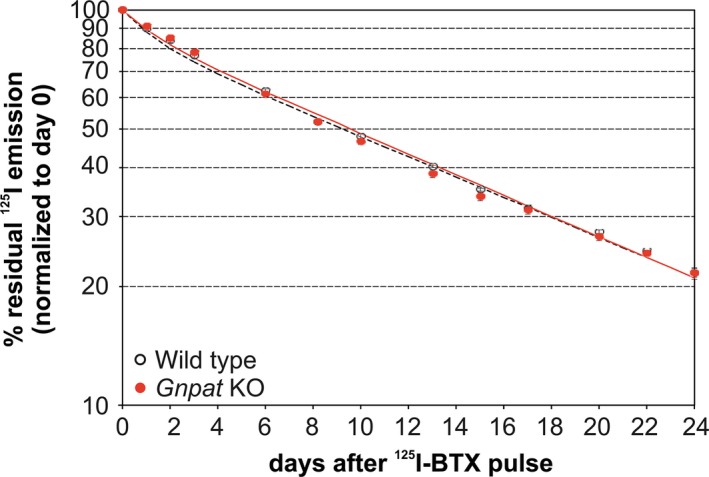
Normal acetylcholine receptor (AChR) stability in ether lipid‐deficient mice as indicated by *in vivo* radioligand binding. Adult WT (*n* = 4 limbs derived from two mice) and *Gnpat* KO (*n* = 6 limbs derived from three mice) mice were injected intramuscularly (*tibialis anterior* muscle) with radioactively labeled α‐BTX and the loss of radioactivity was monitored over 24 days. Data are presented as means (circles) ± SEM and the curves were fitted as described previously (Strack *et al*. 2011). Statistical analysis using two‐tailed Student's *t*‐tests did not reveal significant differences at any time point. Thus, no correction for the repeated measurements was performed.

Because ether lipids, particularly plasmalogens, shape membrane structure and fluidity, membrane proteins like AChRs are likely to be influenced by alterations in membrane lipid composition evoked by the lack of ether lipids. Particularly lipid rafts, which provide the lipid environment for AChR clustering (Pato *et al*. 2008; Stetzkowski‐Marden *et al*. 2006), could be negatively affected by ether lipid deficiency. We, therefore, hypothesized that the absence of ether lipids in *Gnpat* KO mice impairs the stability of AChR clusters. In order to address this question, we labeled AChRs in the *tibialis anterior* muscles of WT and *Gnpat* KO mice by injecting ^125^I‐BTX, which binds irreversibly to muscle‐type AChRs, and studied the loss of radioactive emission as a measure of AChR turnover over time. Remarkably, we obtained almost perfectly identical decay curves for both genotypes (Fig. 6) demonstrating similar lifetimes of AChRs in WT and *Gnpat* KO mice. These results were further confirmed by fluorescence labeling of different AChR subsets: surface receptors were stained using Alexa Fluor 647‐coupled BTX (‘old receptors’). Ten days later, newly generated receptors were stained by Alexa Fluor 555‐coupled BTX (‘new receptors’) and the ratio between new and old receptors was subsequently evaluated as a measure of AChR stability by *in vivo* confocal microscopy. Similarly, as found in the radioligand assay, also by this method *Gnpat* KO mice did not differ from WT controls (Fig. S4).

### Electrophysiological abnormalities are observed at ether lipid‐deficient NMJs

**Figure 7:**
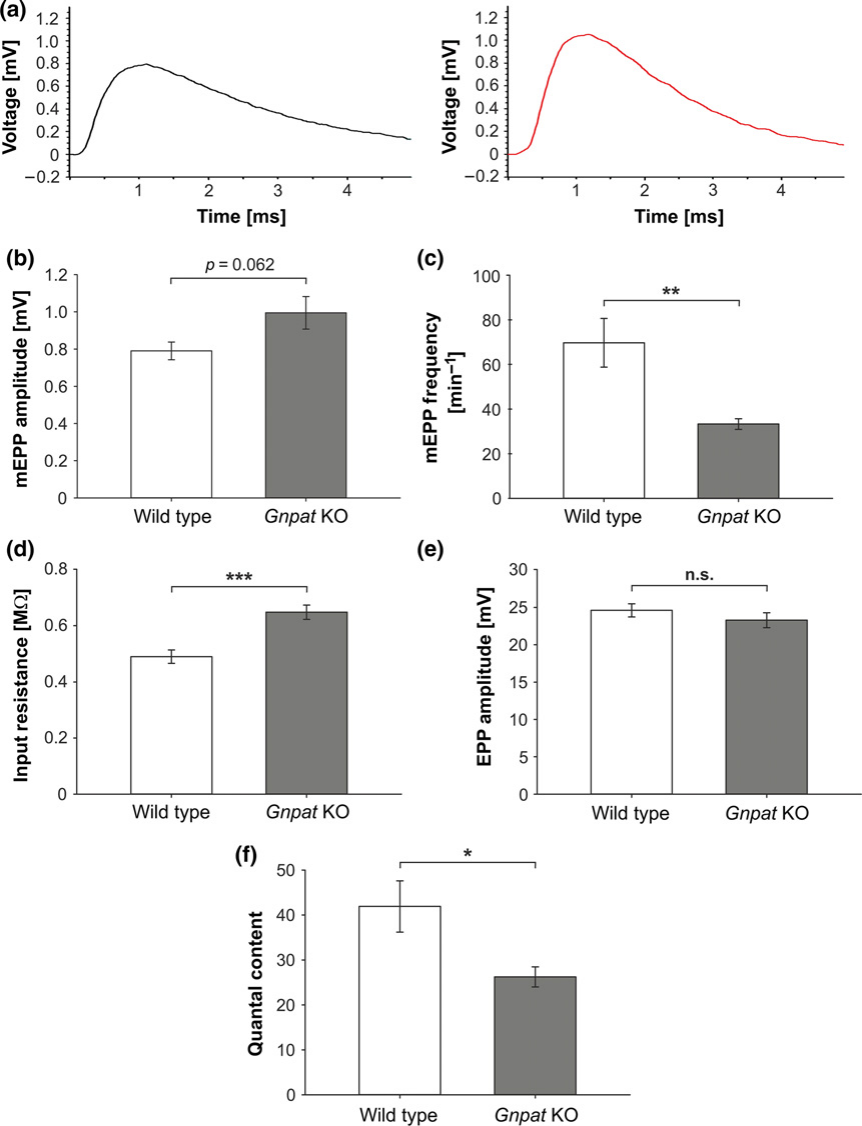
Increased amplitude and strongly reduced frequency of mEPPs but normal evoked potentials at diaphragm muscles of *Gnpat* KO mice. Miniature end plate potentials (mEPPs), reflecting spontaneous vesicle fusion, were recorded from diaphragms of adult WT (left panel) and *Gnpat* KO (right panel) mice; representative traces for each genotype are shown in (a). The mEPP amplitude (b), mEPP frequency (c), and input resistance (d) (b–d: WT, *n* = 7 mice, 88 fibers total; *Gnpat* KO, *n* = 7 mice, 118 fibers total) as well as the amplitude of end plate potentials (EPPs) in response to 1 Hz pulses (WT, *n* = 5 mice, 50 fibers total; *Gnpat* KO, *n* = 7 mice, 86 fibers total) (e) and quantal content (the ratio between end plate currents (EPC) and mEPC; WT, *n* = 5 mice, 43 fibers total; *Gnpat* KO, *n* = 7 mice, 86 fibers total) (f) are presented as means ± SEM. ***p <* *0.001, **p <* *0.01, *p <* *0.05, *n.s*., not significant (two‐tailed Student's *t*‐test).

Based on the morphological abnormalities that we observed in NMJs of *Gnpat* KO mice, we evaluated functional consequences by recording neuromuscular transmission in phrenic nerve‐diaphragm explants dissected from adult WT and *Gnpat* KO mice. We detected, by trend, increased amplitudes of miniature end plate potentials (mEPPs) in *Gnpat* KO compared with WT muscles (Fig. 7a and b). These were accompanied by significantly increased mEPC amplitudes (means ± SEM: WT (*n* = 7 mice, 64 fibers total): 2.338 ± 0.109 nA; *Gnpat* KO (*n* = 7 mice, 85 fibers total): 3.031 ± 0.196 nA; *p *=* *0.009 [two‐tailed Student's *t*‐test]). Rise times of mEPPs did not differ significantly between the genotypes (means ± SEM: WT (*n* = 5 mice, 47 fibers total): 0.314 ± 0.019 ms; *Gnpat* KO (*n* = 5 mice, 63 fibers total): 0.319 ± 0.007 ms; *p *=* *0.829 [two‐tailed Student's *t*‐test]). In addition, we detected a strikingly decreased frequency of mEPPs in fibers derived from *Gnpat* KO mice, demonstrating a reduced number of spontaneous vesicle fusion events at ether lipid‐deficient NMJs (Fig. 7c). Interestingly, in all our recordings, we observed a strong and highly significant increase of about 30% in input resistance in *Gnpat* KO preparations (Fig. 7d), possibly reflecting changes in membrane properties caused by ether lipid deficiency.

To evoke synaptic transmission in response to a pre‐synaptic stimulus, we applied 1 Hz pulses to the phrenic nerve and measured the resulting end plate potentials (EPPs). Remarkably, the amplitude of EPPs did not differ between WT and *Gnpat* KO diaphragm NMJs (Fig. 7e). From the current flows (EPC and mEPC) accompanying EPPs and mEPPs, we calculated the quantal content, a measure of the number of vesicles released upon a pre‐synaptic stimulation event. In line with the observed increase in mEPP amplitude but normal EPPs, the quantal content was significantly reduced in nerve‐muscle preparations derived from *Gnpat* KO mice (Fig. 7f), arguing that vesicle fusion might be impaired as a consequence of ether lipid deficiency. Rise times [means ± SEM: WT (*n* = 5 mice, 52 fibers total): 0.356 ± 0.023 ms; *Gnpat* KO (*n* = 7 mice, 86 fibers total): 0.399 ± 0.020 ms; *p *=* *0.187 (two‐tailed Student's *t*‐test)] and decay times [means ± SEM: WT (*n* = 5 mice, 52 fibers total): 2.558 ± 0.224 ms; *Gnpat* KO (*n* = 7 mice, 86 fibers total): 2.899 ± 0.173 ms; *p *=* *0.249 (two‐tailed Student's *t*‐test)] of EPPs as well as the time constant tau [means ± SEM: WT (*n* = 5 mice, 53 fibers total): 1.723 ± 0.160 ms; *Gnpat* KO (*n* = 7 mice, 84 fibers total): 1.888 ± 0.101 ms; *p *=* *0.381 (two‐tailed Student's *t*‐test)] did not differ significantly between the genotypes. Also, there was no significant difference in capacitance of muscle fibers [means ± SEM: WT (*n* = 5 mice, total 49 fibers): 3.077 ± 0.293 μF/cm^2^; *Gnpat* KO (*n* = 5 mice, total 63 fibers): 2.623 ± 0.280 μF/cm^2^; *p *=* *0.295 (two‐tailed Student's *t*‐test)]. Finally, the response to repetitive stimulation was determined by applying pulses of 5 Hz. The decrement from the first to the 25th recorded EPP was determined for each fiber; no difference was detected between WT and *Gnpat* KO NMJs in this parameter [mean decrement ± SEM: WT (*n* = 5 mice, total 45 fibers): 19.50 ± 1.93%; *Gnpat* KO (*n* = 7 mice, 78 fibers): 15.24 ± 1.21%; *p *=* *0.076 (two‐tailed Student's *t*‐test)].

## Discussion

Because of the poor performance of ether lipid‐deficient mice in tests assessing motor coordination and muscle strength, we suspected impairments at the level of the NMJ in these mice. Indeed, morphological analysis of developing and adult NMJs revealed several abnormalities in *Gnpat* KO mice in comparison with WT littermates. The most striking feature was the defasciculated appearance of the main axons innervating the fetal diaphragm as well as a strongly increased area covered by nerve terminals leading to an overall widening of the total endplate zone (Fig. 4). This NMJ phenotype persisted throughout embryonic development indicating a permanent defect of NMJ morphology.

Development of the mammalian NMJ is a highly complex process, which requires the exchange of molecules between pre‐ and post‐synaptic compartments as well as auxiliary cell types like Schwann cells. Given the numerous signal transduction processes involved in NMJ maturation, lipid rafts are probable candidates contributing to the observed alterations in *Gnpat*‐deficient mice, as these small membrane domains serve as organizing platforms for signaling events (Simons and Toomre 2000). Plasmalogens have been identified as constituents of lipid rafts (Pike *et al*. 2002), but their exact role and how ether phospholipid deficiency affects the stability and function of rafts have not yet been established. In lipid raft fractions isolated from Chinese hamster ovary (CHO) cells with a deficiency in either alkyl‐dihydroxyacetone phosphate synthase, catalyzing the second step in ether lipid biosynthesis (Fig. 1), or peroxisome biogenesis, detergent resistance and lipid composition were normal (Honsho *et al*. 2008). However, the protein and lipid content of lipid rafts can vary considerably depending on their functional context (Pike 2004; Levental *et al*. 2011). Thus, disturbances of phospholipid homeostasis may be more devastating in the increased complexity of a mammalian organism than in cultured cells. This is also underlined by the observation that in the brain of *Gnpat* KO mice, the properties of lipid raft domains and proteins associated with them appear altered (Rodemer *et al*. 2003). The participation of lipid rafts in NMJ maturation have been reported repeatedly; for example, the characteristic clustering of AChRs has been suggested to require rafts (Zhu *et al*. 2006; Stetzkowski‐Marden *et al*. 2006; Campagna and Fallon 2006; Bezakova and Ruegg 2003). Also, agrin, the master organizer of NMJ development, regulates the immunological synapse via modulation of lipid rafts in lymphocytes (Khan *et al*. 2001). Indeed, our *in vitro* study of AChR clustering as well as the quantification of clusters *in vivo* (Fig. 5 and Fig. S3) suggest an impairment in cluster formation upon ether lipid deficiency and it is tempting to speculate that lipid rafts play a central role in the explanation for these findings. A limitation for our clustering experiments is the low number of stable cell lines that we obtained, thus some caution is warranted in the interpretation of these data.

In *Drosophila* it has been shown that the regulation of NMJ maturation involves the AKT signaling pathway (Natarajan *et al*. 2013). In the peripheral nervous system of *Gnpat* KO mice, a deficiency in the phosphorylation of AKT has been observed and was attributed to inefficient recruitment of AKT to the plasma membrane, where the phosphorylation occurs (da Silva *et al*. 2014). Accordingly, this defect might also have an influence on NMJ development. However, the amplitudes of mEPPs recorded from NMJs in flies with a genetically induced AKT phosphorylation deficiency were lower than control values (Natarajan *et al*. 2013) arguing against a defective AKT pathway as the sole explanation for NMJ abnormalities in ether lipid‐deficient mice, which in our electrophysiological studies showed a trend toward elevated mEPP amplitudes. In line with our observations in *Gnpat* KO mice, the AKT mutant flies showed a reduced frequency of mEPPs. Ether lipid deficiency could also impair signaling processes at the level of GPI‐anchored proteins. Prominent examples of GPI‐anchored proteins with designated roles in NMJ development include the matrix metalloproteinase regulator reversion‐inducing‐cysteine‐rich protein with Kazal motifs (RECK) (Kawashima *et al*. 2008) or the receptor tyrosine kinase ligand Ephrin A‐2 (Lai *et al*. 2001), for which a role in the stabilization of the post‐synaptic apparatus at the NMJ is discussed (Lai and Ip 2003). The GPI anchor serves as a sorting determinant for the integration into lipid rafts (Brown and Rose 1992; Benting *et al*. 1999); and the lipid moiety of the anchor itself partly depends on ether lipid biosynthesis (Kanzawa *et al*. 2009). It still remains unclear, whether the absence of ether lipids impairs the function of all or certain GPI‐anchored proteins (Wanders and Brites 2010). Apparently, the activity of GPI‐anchored proteins is not fully abolished, but compensatory changes in the levels of these proteins have been demonstrated in cells from human ether lipid‐deficient patients (Kanzawa *et al*. 2012).

Comparisons to other genetically modified mouse models with disturbances in NMJ development or neuromuscular transmission could provide insight into the molecular causes of the NMJ phenotype observed in *Gnpat* KO mice. For example, mice with a complete inability to form muscle‐type AChRs (An *et al*. 2010) or a deficiency in choline acetyltransferase (Misgeld *et al*. 2002), as well as agrin KO mice (Gautam *et al*. 1996) show abnormal nervous innervation of muscles. In particular, the former two mutants display extensive motor nerve branching and a broadened end plate area, reminiscent of our findings in *Gnpat* KO mice. Elaborate studies in these mice have revealed a complex regulation underlying pre‐ and post‐synaptic specialization at the NMJ, which is not yet fully understood. Ether lipid deficiency might modulate this network, for example, by weakening signaling cascades like MAP kinase pathways, described to be activated by certain ether lipids (Liliom *et al*. 1998), or the AKT pathway (da Silva *et al*. 2014). Phenotypically, this could result in the morphological changes detected in the diaphragm of ether lipid‐deficient mice.

We also examined the morphological characteristics of AChR clusters in different skeletal muscles of adult mice. There was a consistent trend toward smaller clusters, as judged by volume as well as surface area, in all muscles analyzed of *Gnpat* KO mice. However, the differences reached statistical significance only in *soleus* muscle, and, for the surface area, in *gastrocnemius* muscle. *Soleus* muscle is a typical example of a muscle consisting of slow‐twitch fibers, whereas *tibialis anterior* and *extensor digitorum longus* muscles can be classified as fast‐twitch muscles. Interestingly, the two fiber types have been suggested to differ in the head group composition of plasmalogens and, moderately, also in their plasmalogen content (Horrocks 1972; Masoro *et al*. 1966). Remarkably, the *gastrocnemius* muscle constitutes an intermediate type containing both types of fibers. The fact that in this muscle, a significantly smaller surface area was found in *Gnpat* KO mice might indicate that indeed slow‐twitch fibers are more affected than fast‐twitch fibers. A slight impairment in the clustering process evoked by ether lipid deficiency, for example, via destabilization of lipid rafts (as described above) or other interference with the signaling machinery involved in clustering, might explain our findings.

In order to evaluate the functional consequences of ether lipid deficiency for synaptic transmission at the NMJ, we conducted electrophysiological recordings from adult diaphragm muscles *in vitro*. Intriguingly, we detected a strikingly reduced rate of spontaneous vesicle fusion, as indicated by the reduced number of mEPPs in *Gnpat* KO mice. The higher mean amplitude of the mEPPs did not reach statistical significance, whereas that of mEPCs was clearly increased. However, the amplitude of evoked potentials (EPPs) was not affected. These changes were accompanied by a considerable elevation in input resistance likely reflecting altered biophysical properties evoked by the depletion of ether lipids and compensatory changes in lipid composition (Dorninger *et al*. 2015). The proposed involvement of plasmalogens in membrane fusion processes as well as their fast turnover in gray matter has led to suggestions of a role in neurotransmission and related events (Lohner 1996; Han 2005; Farooqui and Horrocks 2001; Rosenberger *et al*. 2002). In addition, plasmalogens are major constituents of pre‐synaptic membranes (Hofteig *et al*. 1985) and synaptic vesicles (Takamori *et al*. 2006); and previous studies using synaptosomes derived from *Gnpat* KO mice supported the hypothesis of impaired vesicular neurotransmitter release under conditions of ether lipid deficiency (Brodde *et al*. 2012). In our current hypothetical model, an impairment of fusion and constriction events because of the lack of plasmalogens and the resulting compensatory changes (Dorninger *et al*. 2015) in vesicular and synaptic membranes would account for the majority of our findings. For example, a reduced rate of membrane fusion is a plausible explanation for the decreased frequency of mEPPs as well as for the reduced number of vesicles released at each stimulated event (quantal content) in ether lipid‐deficient mice. Concurrently, when a synaptic vesicle fuses with the pre‐synaptic membrane, impaired membrane constriction might lead to the release of a large amount of vesicular content instead of a ‘kiss‐and‐run’‐like mode, which like in the central nervous system has been proposed to also occur at the NMJ (Verstreken *et al*. 2002), thus accounting for the elevation in mEPP and mEPC amplitude. Interestingly, altered mEPP properties, particularly reduced frequency of spontaneous vesicle fusion, have been described in many mouse models with a deficiency in proteins involved in the signaling pathways regulating NMJ development and AChR clustering (Shi *et al*. 2010; Li *et al*. 2008; Barik *et al*. 2014). However, in all of these mice, reduced mEPP frequency goes along with decreased mEPP amplitude. In contrast, an increase in mEPP amplitude has been described in relatively few animal models. One remarkable example is mice with a deficiency in SNAP‐25, in which exocytosis of synaptic vesicles is impaired and EPPs cannot be evoked; however, mEPPs were recorded at normal frequency but with considerably increased amplitude (Washbourne *et al*. 2002). The authors proposed reduced activity of acetylcholinesterase as an explanation for the elevated amplitude of mEPPs rather than compromised exo‐ and endocytosis, as hypothesized in our model.

Alternative to these considerations, which focus on a pre‐synaptic deficit upon ether lipid deficiency, our findings could also originate from post‐synaptic alterations. Our data demonstrate a considerable increase in input resistance that might be directly (e.g., by changes in membrane properties because of altered lipid composition) or indirectly (e.g., by altered muscle fiber size) related to ether lipid deficiency. Increased input resistance, as also seen in *Gnpat* KO mice, has been associated with larger mEPP amplitude in some studies (Katz and Thesleff 1957), but such a correlation has been doubted by others (Gage and McBurney 1973). Furthermore, an inverse relationship between quantal content and mEPP amplitude (or quantal size) has been proposed before (Plomp *et al*. 1992; DiAntonio *et al*. 1999; Fong *et al*. 2010) and would imply that the reduced quantal content at ether lipid‐deficient NMJs is an adaptive response resulting from the increased mEPP and mEPC amplitude.

A recent paper describes phenotypic aberrations in the peripheral nervous system of mice carrying a homozygous inactivating mutation in PEX10 (*Pex10* ^*CY/CY*^), a mouse model of a peroxisome biogenesis disorder (Hanson *et al*. 2014). Next to defects in Schwann cell morphology and axon integrity, these mice display morphological alterations at their NMJs, in particular, a decreased colocalization of pre‐ and post‐synaptic markers – a phenomenon that we did not encounter in *Gnpat* KO mice, indicating that the NMJ phenotype of *Pex10* ^*CY/CY*^ mice is not because of the lack of ether lipids alone. However, similar to our *Gnpat* KO mice with isolated ether lipid deficiency, abnormal axonal growth was identified in fetal *Pex10* ^*CY/CY*^ diaphragms (Hanson *et al*. 2014). Intriguingly, electrophysiological recordings in these diaphragms indicated normal shape and number of mEPPs but reduced amplitudes of EPPs, which is in sharp contrast to our results in *Gnpat* KO mice. One probable determinant for these seemingly conflicting findings is the age of experimental animals. Whereas we used adult mice for our electrophysiological studies, the corresponding experiments in *Pex10* ^*CY/CY*^ animals involved fetal stages. Some of the phenotypic aberrations in ether lipid‐deficient mice manifest with advanced age; thus, it is conceivable that the defects in neuromuscular transmission that we observed are not yet present in developing *Gnpat* KO mice. Accordingly, the neuromuscular phenotype in fetal peroxisome‐deficient mice might be independent of ether lipids and instead be evoked by one of numerous other metabolic defects resulting from generalized peroxisomal dysfunction.

Since poor performance in the balance beam, rotarod, or muscle strength tasks have frequently been associated with neuromuscular dysfunction (Gomez *et al*. 1997; Pelkonen and Yavich 2011; Shi *et al*. 2010), it is tempting to ascribe these deficits to the NMJ abnormalities of ether lipid‐deficient mice. However, from our electrophysiological data (particularly the normal EPP amplitude and the unchanged decrement after high frequency pulses), we conclude that there is no major impairment of neuromuscular transmission in these animals. More subtle deficits might become detectable in more detailed analyses as described elsewhere (Sandrock *et al*. 1997). Given the widespread effects of ether lipid deficiency on the mammalian organism, it is generally problematic to assign a phenotypic feature like motor dysfunction to a particular molecular alteration. We consider it likely that motor impairments in *Gnpat* KO mice result from the combined presence of cerebellar deficits (Teigler *et al*. 2009) and peripheral myelination defects (da Silva *et al*. 2014), which may be modulated by some of the abnormalities in NMJ development and transmission as reported here.

Taken together, our results indicate that ether lipid deficiency affects both structure and function of the NMJ. Based on the present findings, the lack of ether phospholipids apparently modulates specific NMJ properties rather than abolishing transmission at the NMJ.

## Supporting information


**Figure S1.** No association between the scores achieved in the weights test and body weight.
**Figure S2.** AChR clusters in fetal diaphragms of WT and *Gnpat* KO mice.
**Figure S3.** AChR clusters in skeletal muscles of adult WT and *Gnpat* KO mice.
**Figure S4.** *In vivo* ligand binding for fluorescence‐based evaluation of AChR stability in WT and *Gnpat* KO mice.
**Table S1.** Scoring system for the evaluation of balance beam performance.
