# Supplementary material for: Reduced muscle strength in ether lipid‐deficient mice is accompanied by altered development and function of the neuromuscular junction
Source: J Neurochem. 2017 Sep 25;143(5):569–83. doi: 10.1111/jnc.14082 (PMC5725694; doi:10.1111/jnc.14082)
Supplement: Supplementary file 1 — Figure S1. No association between the scores achieved in the weights test and body weight. Figure S2. AChR clusters in fetal diaphragms of WT and Gnpat KO mice. Figure S3. AChR clusters in skeletal muscles of adult WT and Gnpat KO mice. Figure S4. In vivo ligand binding for fluorescence‐based evaluation of AChR stability in WT and Gnpat KO mice. Table S1. Scoring system for the evaluation of balance beam performance. [file JNC-143-569-s001.pdf]

Supporting Information

Reduced muscle strength in ether lipid-deficient mice is accompanied by altered development and function of the neuromuscular junction

Fabian Dorninger, Ruth Herbst, Bojana Kravic, Bahar Z. Camurdanoglu, Igor Macinkovic, Gerhard Zeitler, Sonja Forss-Petter, Siegfried Strack, Muzamil Majid Khan, Hans R. Waterham, Rüdiger Rudolf, Said Hashemolhosseini, Johannes Berger

Supporting Methods

Determination of plasmalogen levels

Muscle tissue was isolated from the hind limbs of *Gnpat* KO and WT mice (*gastrocnemius* and *quadriceps femoris*) and homogenized using a tissue homogenizer (Polytron PT3100 equipped with a PT-DA 3012/2 S aggregate, Kinematica) for 10 sec at 15,000 rpm in PBS. After centrifugation (1000*xg*, 4 °C), additional homogenization of the pellet was performed with 10 strokes using a glass-Teflon tissue grinder (Potter-Elvehjem homogenizer). The supernatants after centrifugation (1000*xg*, 4 °C) were combined and plasmalogen levels determined by detection of dimethylacetals (DMA) after acidic methanolysis as described previously (Dacremont & Vincent 1995).

Analysis of motor performance

To avoid variability resulting from sex differences, only male mice at the age of 3-8.5 months (age-matched *Gnpat* KO and WT mice; littermates, when possible) were used for behavioral studies. All tests were conducted in the evening (at the end of the light phase) under conditions of dim light.

Rotarod. The rotarod tests were performed as described previously (Dumser *et al.* 2007). Briefly, mice were placed in separate lanes on the slowly rotating (4 rpm) cylinder of an accelerating rotarod apparatus (Ugo Basile). After 30 to 60 sec for familiarization, the timer and accelerating mode (4-40 rpm in 300 sec) were started and left running at maximum speed until 500 sec from the start. The latency to fall (in sec) was stopped automatically. Two rotations of mice clinging to the cylinder were assessed as a fall. A 3-day training/test scheme was applied: Mice were trained in three trials on two consecutive days followed by the test in four consecutive trials on the third day with suitable resting periods between the trials. The mean value of the best three trials on the test day was calculated for each mouse and used for statistical analysis.

Balance beam. Mice were placed on the balance beam, a 60 cm long wooden rod above the padded floor. The horizontal midline was marked on the beam as reference for hind limb placement, with slips indicating motor coordination deficits. Mice were first placed in the middle of the rod and allowed to explore their surroundings and were then relocated to one end of the bar. Movement across the beam to a platform was encouraged and the performance was evaluated according to a subjective scoring system (Table S1) modified from (Carter *et al.* 2001) by two investigators blinded to the genotype of the mice (although the genotype might be overt in most cases due to the phenotypical alterations of *Gnpat* KO mice). Each mouse was allowed three consecutive trials interrupted by suitable resting periods. The best score (usually reached in the third trial) was used for statistical analysis.

Analysis of muscle strength

Two different tests of muscle strength were applied as described previously (Deacon 2013) with slight modifications.

Weights test. Mice were held near the base of their tail and allowed to grasp a wire scouring pad, to which weights were attached. Subsequently, they were lifted and the time until releasing the weight pad recorded. Different weights (33 g, 43 g, 53 g, 63 g, 73 g and 83 g) were tested in ascending order. The ability to lift a weight of 17 g (corresponding to the wire scouring pad alone) was a prerequisite for entering the experiment (accomplished by all mice tested). The ability to lift the weight for 3 sec was recorded as a positive trial and the mouse was allowed to proceed to the next weight. After three negative trials (<3 sec) with the same weight, the mouse was taken out of the experiment, but still exposed to the next heavier weight in order to exclude lack of motivation. Scores were calculated as the sum of the ranked coefficients for the weights (1 for 33 g, 2 for 43 g, etc.) multiplied by the time held (3 sec for a positive trial or less if released earlier). Suitable resting periods (minimum 5 min) were granted between the trials.

Inverted screen. Mice were placed on a mesh wire grid about 50 cm above a cage filled with bedding. After few sec for familiarization, the screen was inverted and the latency to fall or a maximum time of 90 sec recorded. The test was conducted on two days within one week with two trials per day. The average of all trials for each mouse was calculated for statistical analysis.

Monitoring neural transmission by electrophysiology

Recordings at the NMJ were mainly performed as described earlier (Kravic *et al.* 2016). The phrenic nerve of diaphragm was maintained in Liley's solution gassed with 95% O_2/5% CO_2 (Liley 1956). The preparation was placed on the stage of a Zeiss Axio-Examiner Z1 microscope fitted with incident light fluorescence illumination with filters for red fluorescing fluorophore (Carl Zeiss MicroImaging GmbH). At the beginning of the experiment the compound muscle action potential (cMAP) was recorded. The electrode was positioned such that the latency of the major negative peak was minimized. The electrode was then positioned 100 µm above the surface of the muscle. AChRs were labeled by adding rhodamine-α-BTX (5 nM, Molecular Devices). After labeling, preparations were exposed to µ-conotoxin GIIIB (µ-CTX, 2 µM, Peptide Institute) to block muscle action potentials, so that end plate potentials (EPPs) could be recorded (Rogozhin *et al.* 2008, Plomp *et al.* 1992).

Two intracellular electrodes were inserted within 50 µm of the NMJs under visual inspection (Rogozhin *et al.* 2008). Current was passed through one electrode to maintain the membrane potential within 2 mV of -75 mV, while voltage transients were recorded with the other. Signals were amplified by an Axoclamp 900A and digitized at 40 kHz by a Digidata 1440A under the control of pCLAMP 10 (Molecular Devices). Voltage records were filtered at 3 kHz and current records at 1 kHz (8-pole Bessel filter). Current transients were recorded using the two-electrode voltage-clamp facility of the Axoclamp 900A. Clamp gains were usually 300-1,000. At most NMJs,

50-100 spontaneous quantal events were recorded during a period of 1 min. A similar number of evoked events was recorded during nerve stimulation at 1 Hz. Records were analyzed using pCLAMP 10. The events recorded from each NMJ were averaged.

Morphologic examination of NMJs

For studies of NMJ development, pregnant mice were sacrificed by CO_2 inhalation. Fetuses were retrieved by Cesarean section at gestational day E14.5, E16.5 or E18.5, decapitated and diaphragms dissected and fixed in 1% paraformaldehyde (PFA) in phosphate-buffered saline (PBS) overnight. After removal of connective tissue, diaphragms were washed in PBS and treated with 0.1% glycine/PBS for 1 h; washed again and permeabilized with 0.5% Triton X-100/PBS for 30-45 min; washed again and blocked with 5% bovine serum albumin (BSA), 0.1% Triton X-100 in PBS for 2-4 h. The following antibodies were diluted in blocking solution and applied at 4 °C overnight: rabbit α-synaptophysin (Invitrogen; 1:5); rabbit α-neurofilament M (Chemicon; 1:400). After washing, samples were stained with a secondary antibody (Alexa Fluor 488-coupled goat α-rabbit; Invitrogen; 1:500 in blocking solution) together with Alexa Fluor 594-coupled α-bungarotoxin (α-BTX; Invitrogen; 1:500) at 4 °C overnight. Samples were postfixed in 1% PFA/PBS for 1 h and mounted using Vectashield® mounting medium (Vector Laboratories). Fluorescence was visualized using a confocal microscope (TCS SP5, Leica Microsystems). Z stacks were generated and pictures depicted as 3D projections of these stacks obtained by using the corresponding software (Leica Application Suite AF). Further image processing was done using Photoshop (Adobe) and ImageJ (NIH). Quantification of the area covered by nerves and nerve endings was performed similarly as described previously (Li *et al.* 2008). Three regions with defined distance from the phrenic nerve were analyzed in each diaphragm: dorsal, central and ventral. In these regions, the area covered by neurons was traced using the polygon tool of ImageJ and the area of the resulting polygon was calculated automatically. For analysis, data of *Gnpat* KO fetuses were exclusively referred to littermate WT controls in order to ensure identical embryonic staging. Regions were weighted according to their average size in all animals of a certain embryonic stage and the mean area covered by every region was calculated as described above (due to technical issues, not every region was available from all diaphragms). For statistical analysis, all values were normalized to the WT mean of every litter.

For the analysis of mature acetylcholine receptor clusters, adult mice were sacrificed by CO_2 inhalation. Skeletal muscles (*soleus*, *gastrocnemius*, *extensor digitorum longus* and *tibialis anterior* muscles) were dissected and fixed in 2% PFA/PBS for 2 h at 4 °C and kept overnight in 0.5% PFA/PBS. On the next day, muscle bundles were prepared, washed in PBS and stained with rhodamine-coupled BTX (Molecular Devices; 1:2,500 in PBS) for 1 h at 25 °C. Cell nuclei were stained with 4',6-diamidino-2-phenylindole (DAPI; 1:10,000 in PBS) for 5 min. After washing in PBS, muscles were mounted in Mowiol. 3D images of NMJs were taken with EC Plan-NEOFLUAR 409/1.3 oil objective (Zeiss Axio Examiner Z1) at 55 msec exposure time. Images

were deconvoluted and analyzed using 3D deconvolution and 3D measurement modules in AxioVision Software (Zeiss) (Kravic *et al.* 2016).

Supporting References

Carter, R. J., Morton, J. and Dunnett, S. B. (2001) Motor coordination and balance in rodents. *Curr. Protoc. Neurosci.,* **15,** 8.12.1-8.12.14.

Dacremont, G. and Vincent, G. (1995) Assay of plasmalogens and polyunsaturated fatty acids (PUFA) in erythrocytes and fibroblasts. *J. Inherit. Metab. Dis.,* **18 Suppl 1,** 84-89.

Deacon, R. M. (2013) Measuring the strength of mice. *J. Vis. Exp.,* **76,** e2610.

Dumser, M., Bauer, J., Lassmann, H., Berger, J. and Forss-Petter, S. (2007) Lack of adrenoleukodystrophy protein enhances oligodendrocyte disturbance and microglia activation in mice with combined Abcd1/Mag deficiency. *Acta Neuropathol.,* **114,** 573-586.

Kravic, B., Huraskin, D., Frick, A. D. et al. (2016) LAP proteins are localized at the post-synaptic membrane of neuromuscular junctions and appear to modulate synaptic morphology and transmission. *J. Neurochem.,* **139,** 381-395.

Li, X. M., Dong, X. P., Luo, S. W. et al. (2008) Retrograde regulation of motoneuron differentiation by muscle beta-catenin. *Nat. Neurosci.,* **11,** 262-268.

Liley, A. W. (1956) An investigation of spontaneous activity at the neuromuscular junction of the rat. *J. Physiol.,* **132,** 650-666.

Plomp, J. J., van Kempen, G. T. and Molenaar, P. C. (1992) Adaptation of quantal content to decreased postsynaptic sensitivity at single endplates in alpha-bungarotoxin-treated rats. *J. Physiol.,* **458,** 487-499.

Rogozhin, A. A., Pang, K. K., Bukharaeva, E., Young, C. and Slater, C. R. (2008) Recovery of mouse neuromuscular junctions from single and repeated injections of botulinum neurotoxin A. *J. Physiol.,* **586,** 3163-3182.

Table S1: Scoring system for the evaluation of balance beam performance

Score	Description
**1**	Performs task without problem, runs swiftly across the beam stepping with all four paws on top quarter of the rod
**1.5**	Performs task without problem, runs hesitantly across the beam stepping with all four paws on top quarter of the rod
**2**	Performs task, slipping occasionally with hind paws below top quarter of the rod
**2.5**	Performs task, slipping repeatedly with hind paws below top quarter of the rod
**3**	Performs task, slipping frequently with hind paws below midline of the rod
**3.5**	Performs task poorly, occasionally dragging hind limbs below midline or slipping with front and hind limbs below midline
**4**	Performs task poorly, repeatedly dragging hind limbs below midline or slipping with front and hind limbs below midline
**4.5**	Performs task poorly, frequently dragging hind limbs below midline or slipping with front and hind limbs below midline
**5**	Cannot perform task; falls off the beam

Supporting Figure Legends

Figure S1: *No association between the scores achieved in the weights test and body weight.* Scores obtained in the weights test are plotted against the body weight of the test animal within the cohort of WT (n = 30; left panel) and *Gnpat* KO (n = 31; right panel) mice. Associations were quantified using Pearson's correlation coefficients.

Figure S2: *AChR clusters in fetal diaphragms of WT and Gnpat KO mice.* (A) Diaphragms from WT and *Gnpat* KO mice (E18.5) were stained with Alexa Fluor 594-coupled α-BTX. Representative confocal images are shown in (A). Note the dispersed appearance of AChR clusters in *Gnpat* KO diaphragms; scale bar = 250 µm. (B) Upon higher magnification, pre- and post-synaptic components of the NMJ (immunofluorescence stainings as in Fig. 4 and in panel A) are colocalized in both genotypes; scale bar = 50 µm; AChR, acetylcholine receptor; NF M, neurofilament M

Figure S3: *AChR clusters in skeletal muscles of adult WT and Gnpat KO mice.* AChR clusters were labeled with rhodamine-coupled α-BTX. Representative images of AChR clusters in *gastrocnemius* muscle of WT and *Gnpat* KO mice are shown in (A); scale bar = 10 µm. Quantifications of cluster volume (B), surface area (C), mean grey value (D) and the number of fragments (E) are presented as means ± SEM (n = 20 clusters per muscle and genotype). ***$p < 0.001$, **$p < 0.01$, *$p < 0.05$ (two-tailed Student's t-test, Bonferroni-Holm correction for the multiple muscles analyzed); SOL, *soleus*; GAS, *gastrocnemius*; EDL, *extensor digitorum longus*; TA, *tibialis anterior*

Figure S4: *In vivo ligand binding for fluorescence-based evaluation of AChR stability in WT and Gnpat KO mice.* AChRs were labeled by intramuscular injection (*tibialis anterior* muscle) of Alexa Fluor 647-coupled α-BTX (green) and, ten days later, with Alexa Fluor 555-coupled α-BTX (red). Subsequently, the ratio between newly formed receptors (stained by Alexa Fluor 555; "new AChR") and "old" AChRs (stained by Alexa Fluor 647) was assessed by *in vivo* fluorescence microscopy. No obvious difference could be detected between WT and *Gnpat* KO mice (n = 2/genotype).

Figure S1

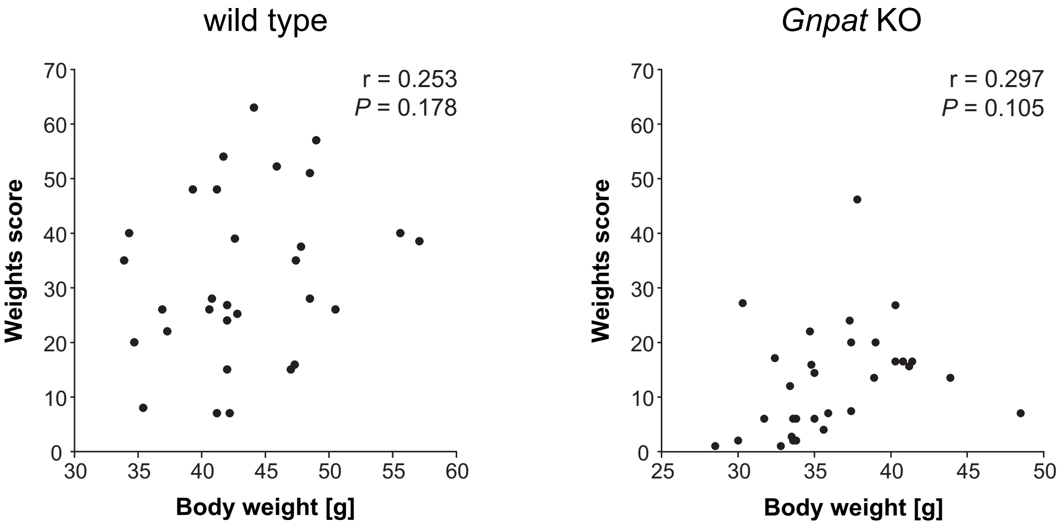
wild type
r = 0.253
P = 0.178
Weights score
Body weight [g]
0
10
20
30
40
50
60
70
30
35
40
45
50
55
60
Gnpat KO
r = 0.297
P = 0.105
Weights score
Body weight [g]
0
10
20
30
40
50
60
70
25
30
35
40
45
50

Figure S2

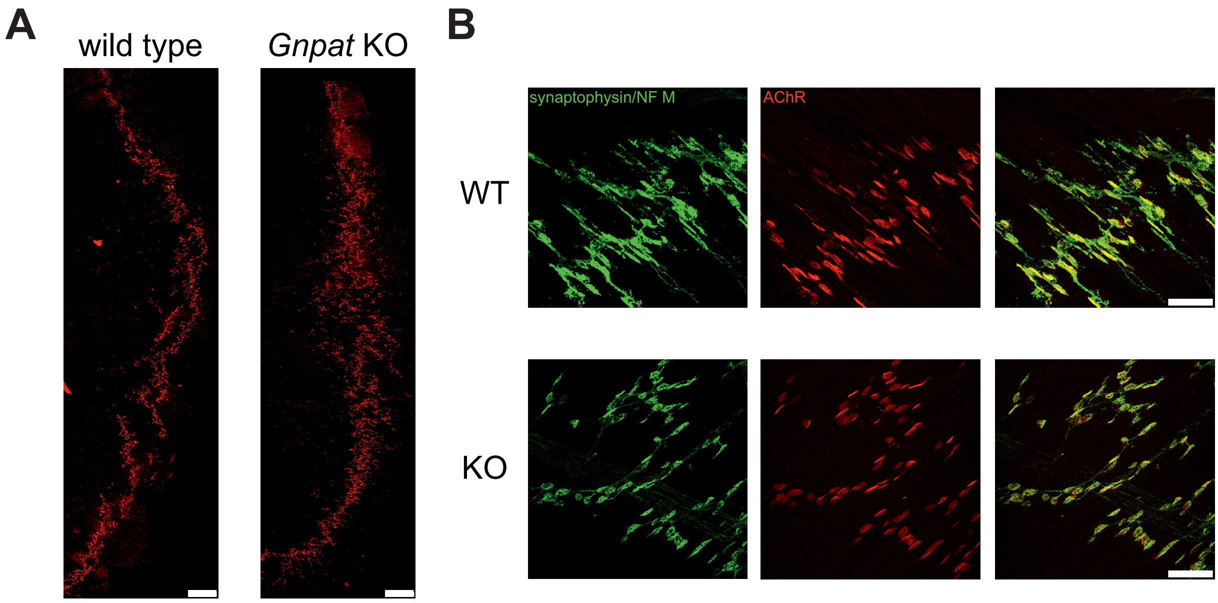

Figure S3

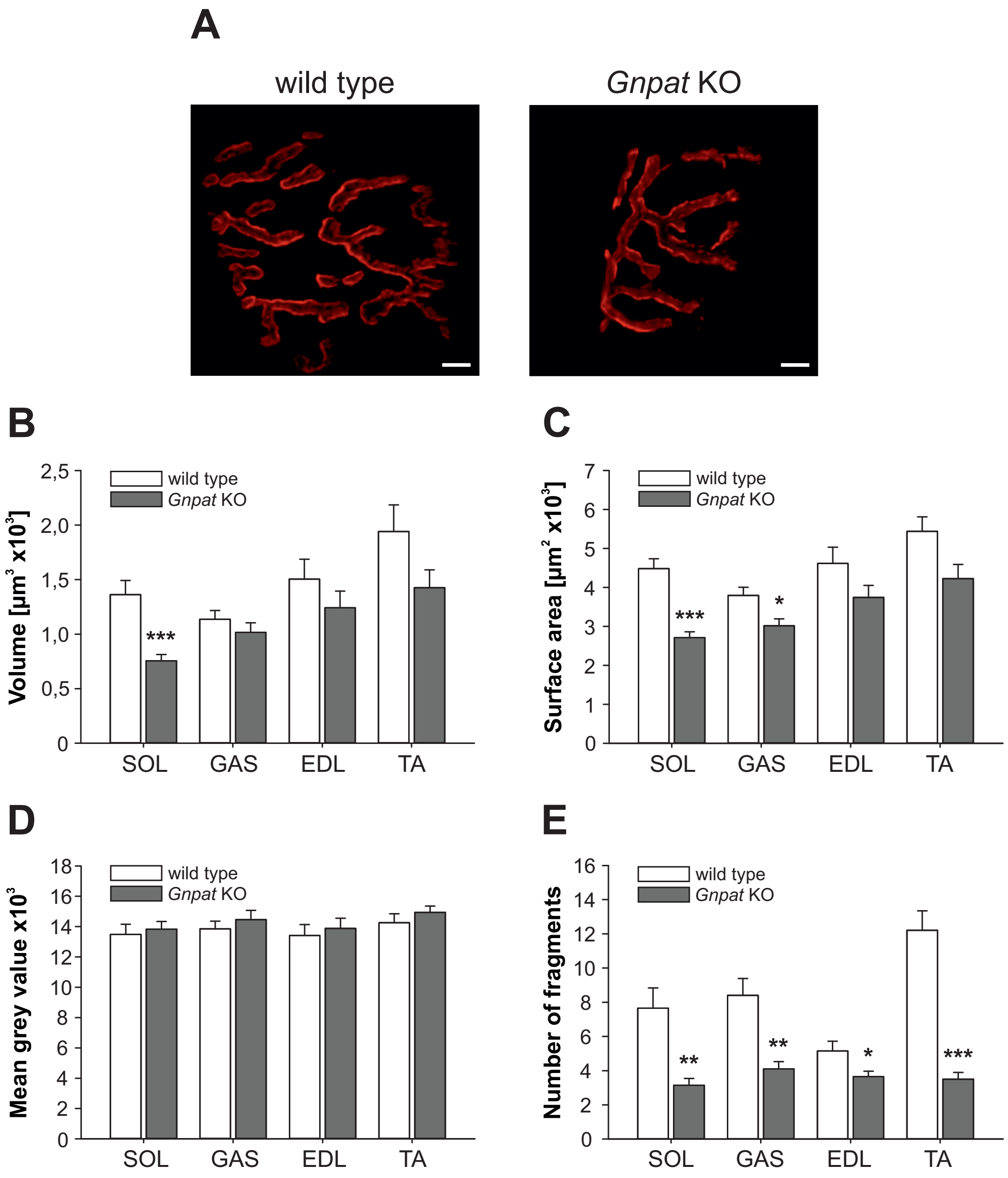

A
wild type
Gnpat KO
B
C
D
E
wild type
Gnpat KO
Volume [µm³ x10³]
Surface area [µm² x10³]
Mean grey value x10³
Number of fragments
2,5
2,0
1,5
1,0
0,5
0
SOL
GAS
EDL
TA
***
*
**

Figure S4

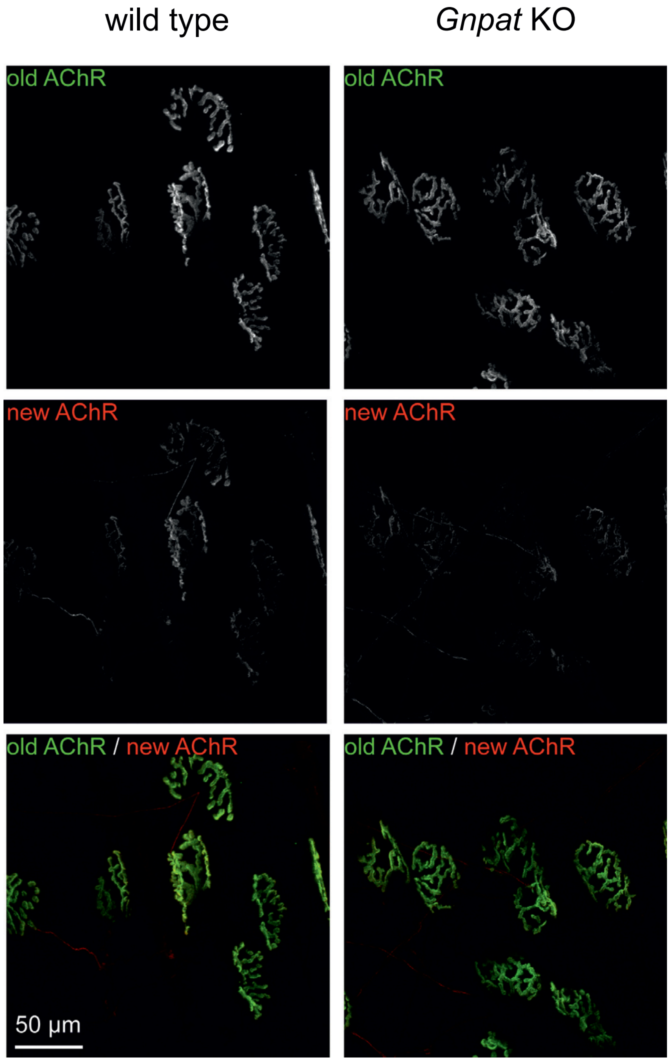
wild type
Gnpat KO
old AChR
old AChR
new AChR
new AChR
old AChR / new AChR
old AChR / new AChR
50 µm
